# Vitamin D Enhanced the Osteogenic Differentiation of Cell Spheroids Composed of Bone Marrow Stem Cells

**DOI:** 10.3390/medicina57111271

**Published:** 2021-11-19

**Authors:** Hyun-Jin Lee, Young-Min Song, Seunghoon Baek, Yoon-Hee Park, Jun-Beom Park

**Affiliations:** 1 Department of Periodontics, College of Medicine, The Catholic University of Korea, Seoul 06591, Korea; 21700035@cmcnu.or.kr (H.-J.L.); 22003897@cmcnu.or.kr (Y.-M.S.); 2 Guidance Dental, Buena Park, CA 90621, USA; mosesbaek@hotmail.com; 3 Ebiogen, #405, Sungsu A1 Center 48 Ttukseom-ro 17-ga-gil, Seongdong-gu, Seoul 04785, Korea; yhpark@e-biogen.com

**Keywords:** cell differentiation, osteogenesis, stem cells, vitamin D

## Abstract

*Background and Objectives:* Vitamin D is a bone modulator widely used in regenerative medicine. This study aimed to analyze the effects of vitamin D on the osteogenic differentiation and mineralization of human mesenchymal stem cells. *Materials and Methods:* Spheroids were fabricated using human bone marrow-derived stem cells, and were cultured in the presence of vitamin D at concentrations of 0, 0.1, 1, 10, and 100 nM. Stem cell spheroids were fabricated and the morphological evaluation was conducted on days 1, 3, 7 and 14. Determination of qualitative cellular viability was performed with Live/Dead Kit assay on days 1 and 7. Quantitative cellular viability was evaluated with Cell Counting Kit-8 on days 1, 3, 7, and 14. To analyze the osteogenic differentiation of cell spheroids, alkaline phosphatase activity assays were performed with commercially available kit on days 7 and 14. Real-time polymerase chain reaction was used to determine the expression levels of RUNX2, BSP, OCN, and COL1A1 on days 7 and 14. *Results:* The stem cells produced well-formed spheroids, and addition of vitamin D did not result in any noticeable changes in the shape. The addition of vitamin D did not significantly change the diameter of the spheroids at 0, 0.1, 1, 10, or 100 nM concentrations. Quantitative cell viability results from days 1, 3, 7 and 14 showed no significant difference between groups (*p* > 0.05). There was significantly higher alkaline phosphatase activity in the 0.1 nM group when compared with the control group on day 14 (*p* < 0.05). Real-time polymerase chain reaction results demonstrated that the mRNA expression levels of RUNX2, OCN, and COL1A1 were significantly increased when vitamin D was added to the culture. *Conclusions:* Based on these findings, we concluded that vitamin D could be applied to the increased osteogenicity of stem cell spheroids.

## 1. Introduction

Vitamin D is a bone modulator widely used in regenerative medicine [1]. Vitamin D also regulates both innate and adaptive immunity, modulates inflammatory cytokine production and blocks antigen-presenting dendritic cell maturation [2]. Several studies described vitamin D’s utility to enhance osteogenesis in primary murine osteoblasts and MC3T3-E1 cell lines [3,4,5]. Replenishing the cell culture medium containing vitamin D induces osteocalcin expression in osteoblasts [6]. Addition of both vitamin D and osteogenic factors resulted in an osteoblast phenotype which expresses alkaline phosphatase activity, secretes osteocalcin, and deposits calcium [6]. Mechanical testing showed that vitamin D induced a stiffer osteosphere compared with control [7]. Vitamin D enhanced cell responses of osteoblasts on the titanium surfaces [8]. Vitamin D is reported to act on osteoblasts through vitamin D receptors and membrane-binding protein [9]. Low dietary intake of vitamin D is reported to be negatively associated with fracture risk [7]. 

Mesenchymal stem cells may be ideal for tissue regeneration because they are highly prolific and have the potential for differentiation into different type of cells [10]. Mesenchymal stem cells can be isolated from a variety of tissues and organs, including bone, fat, periosteums, skeletal muscles and peripheral blood [11]. The aggregation of mesenchymal stem cells into multicellular spheroids resulted in an increase in therapeutic capacity by improving the survival of the stem cells, stemness, angiogenic and anti-inflammatory properties [12]. This method has been proposed as a promising strategy for stem cell therapy [13]. The effects of growth factors on cell survival and osteogenic differentiation of stem cell spheroids have been previously tested, which is of great interest to researchers and clinicians [14]. Short peptides have been reported to play an important role in biological information transfer, transcriptional regulation, and recovery of age-related genetic changes, and some short peptides are reported to promote differentiation of human periodontal ligament stem cells [15]. Platelet-rich fibrin enhances the osteogenic differentiation of human mesenchymal stem cells, and application of platelet-rich fibrin resulted in significant improvements in clinical and radiographic parameters [16,17]. This study aimed to analyze the effects of vitamin D on the osteogenic differentiation and mineralization of cell spheroids composed of human mesenchymal stem cells.

## 2. Materials and Methods

### 2.1. Design of the Present Study 

**Figure 1:**
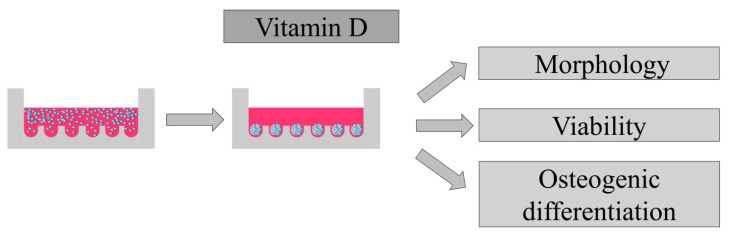
Study flow diagram illustrating the overview.

Figure 1 provides an overview of the current study design. This research protocol has been reviewed and approved by the Institutional Review Board (KC21SASE0225, Approval date: 6 April 2021). We obtained prior consent from the participant. The culture media were changed every two to three days. The cells were grown in an incubator at 37 °C with 95% air and 5% CO_2_.

### 2.2. Fabrication of Stem Cell Spheroids

Human bone marrow-derived mesenchymal stem cells from a male participant (Catholic MASTER Cells) were obtained from the Catholic Institute of Cell Therapy (CIC, Seoul, South Korea) [18]. Stem cells were plated onto silicon elastomer-based concave microwells (StemFIT 3D; MicroFIT, Seongnam-si, Gyeonggi-do, Korea) of 600 µm diameter at a density of 1 × 10^6^ cells/well and cultured in osteogenic media [19]. The medium was replaced with fresh media every two to three day. The final concentrations of vitamin D (1α,25-Dihydroxyvitamin D3; D1530-10UG, Sigma-Aldrich, St. Louis, MO, USA) were 0, 0.1, 1, 10, and 100 nM, respectively. The morphological evaluation was carried out on days 1, 3, 7, and 14 using an inverted microscope (CKX41SF, Olympus Corporation, Tokyo, Japan).

### 2.3. Determination of Qualitative and Quantitative Cell Viability

The qualitative cell viability of cell spheroids cultured in osteogenic media was evaluated with Live/Dead Kit assay (Molecular Probes, Eugene, OR, USA) on days 1 and 7 [20]. These spheroids were incubated at room temperature for 60 min and were observed at ×100 magnification using a confocal laser scanning microscope (LSM800, Carl Zeiss, Germany). Quantitative cell viability test was conducted using Cell Counting Kit-8 (Dojindo, Tokyo, Japan) on days 1, 3, 7, and 14 [21]. 

### 2.4. Evaluation of Alkaline Phosphatase Activity 

Alkaline phosphatase activity levels were used to evaluate osteogenic differentiation using commercially available kit (K412-500, BioVision, Inc., Milpitas, CA, USA) on days 7 and 14 [22]. The absorbance at 405 nm were measured after mixing substrate with cell lysates after incubating for 30 min at 37 °C [21].

### 2.5. Total RNA Extraction and Quantification of RUNX2, BSP, OCN and COL1A1 mRNA by Real-Time Quantitative Polymerase Chain Reaction (qPCR)

Total RNA extraction was performed using a commercially available kit (Thermo Fisher Scientific, Inc., Waltham, MA, USA), according to the manufacturer’s instructions [23]. The quality of RNA was evaluated with a bioanalyzer (Agilent 2100) using a kit (RNA 6000 Nano Chip; Agilent Technologies, Santa Clara, CA, USA), and RNA quantity was evaluated with the ratio of absorbance at 260 nm and 280 nm using a spectrophotometer (ND-2000, Thermo Fisher Scientific, Inc.). RNA was used as reverse transcription template applying reverse transcriptase (SuperScript II; Invitrogen, Carlsbad, CA, USA).

mRNA expression was detected by qPCR on days 7 and 14. We used GenBank to design the sense and antisense primers for PCR. The primer sequences were as follows: RUNX2 (accession No.: NM_001015051.3; forward: 5′-CAGTTCCCAAGCATTTCATCC-3′, reverse: 5′-AGGTGGCTGGATAGTGCATT-3′), BSP (accession No.: NM_004967.4; forward: 5′-CCTCTCCAAATGGTGGGTTT-3′, reverse: 5′-ATTCAACGGTGGTGGTTTTC-3′), OCN (accession No.: NM_199173.6; forward 5′-GGTGCAGAGTCCAGCAAAGG-3′, reverse: 5′-GCGCCTGGGTCTCTTCACTA-3′), COL1A1 (accession No.: NM_000088.4; forward: 5′-TACCCCACTCAGCCCAGTGT-3′, reverse: 5′-CCGAACCAGACATGCCTCTT-3′), and β-actin (accession. No.: NM 001101: forward: 5′-AATGCTTCTAGGCGGACTATGA-3′, reverse: 5′-TTTCTGCGCAAGTTAGGTTTT-3′) [24,25].

### 2.6. Statistical Analysis

All values are presented as mean ± standard deviation. Tests of normality and equality of variances were conducted. Comparisons between the groups were performed by one-way analysis of variance with Tukey’s post hot test. Three technical replicates were evaluated for each analysis. 

## 3. Results

### 3.1. Cell Spheroids of Human Bone Marrow-Derived Mesenchymal Stem Cells

**Figure 2:**
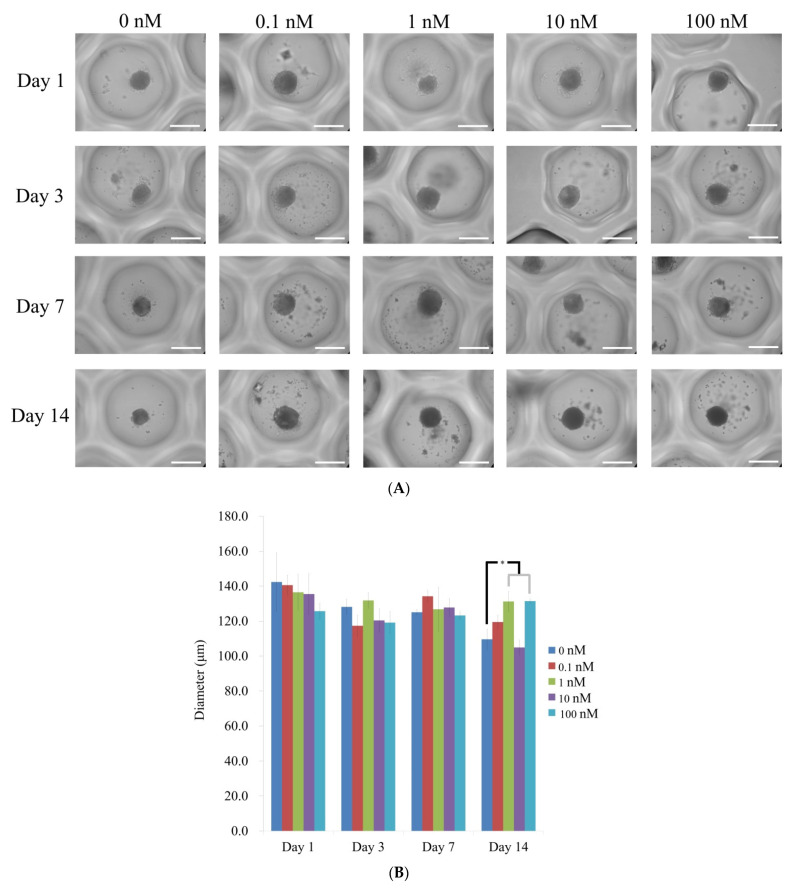
(**A**) The morphologies of stem cell spheroids treated with different concentrations of vitamin D on days 1, 3, 7, and 14. The scale bar represents 200 μm (original magnification ×200). (**B**) The diameters of the stem cell spheroids on days 1, 3, 7, and 14. * *p* < 0.05 vs. time-matched 0 nM group.

The morphology of spheroid treated with vitamin D at final concentrations of 0, 0.1, 1, 10, and 100 nM on days 1, 3, 7 and 14 is shown in Figure 2A. Stem cell spheroids did not show any morphological changes during the 14 days. All stem cell spheroids kept their round figure and maintained their size from day 1 through to day 14. The diameter of the spheroids can be found in Figure 2B. On day 1, the diameters were 142.4 ± 16.8, 140.6 ± 5.7, 136.5 ± 10.3, 135.5 ± 11.8, and 125.7 ± 4.5 µm for vitamin D at 0, 0.1, 1, 10, and 100 nM groups, respectively (*p >* 0.05). On day 3, the diameters for vitamin D at 0, 0.1, 1, 10, and 100 nM concentrations were 128.2 ± 4.3, 117.3 ± 6.1, 131.8 ± 4.5, 120.4 ± 6.8, and 119.1 ± 6.4 µm, respectively (*p >* 0.05). The diameters on day 7 were 125.0 ± 1.5, 134.2 ± 3.4, 126.8 ± 12.7, 127.8 ± 5.2, and 123.2 ± 2.1 for vitamin D at 0, 0.1, 1, 10, and 100 nM groups, respectively (*p >* 0.05). On day 14, the diameters were 109.6 ± 6.0, 119.5 ± 3.9, 131.3 ± 5.8, 104.9 ± 4.3, and 131.4 ± 1.3 µm for vitamin D at 0, 0.1, 1, 10, and 100 nM groups, respectively (*p <* 0.05).

### 3.2. Qualitative Determination and Quantitative Values for Cell Viability

**Figure 3:**
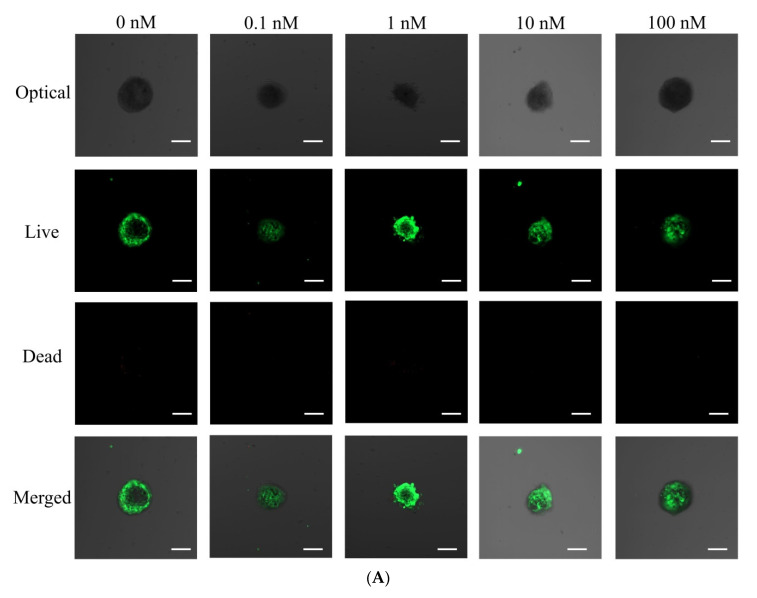

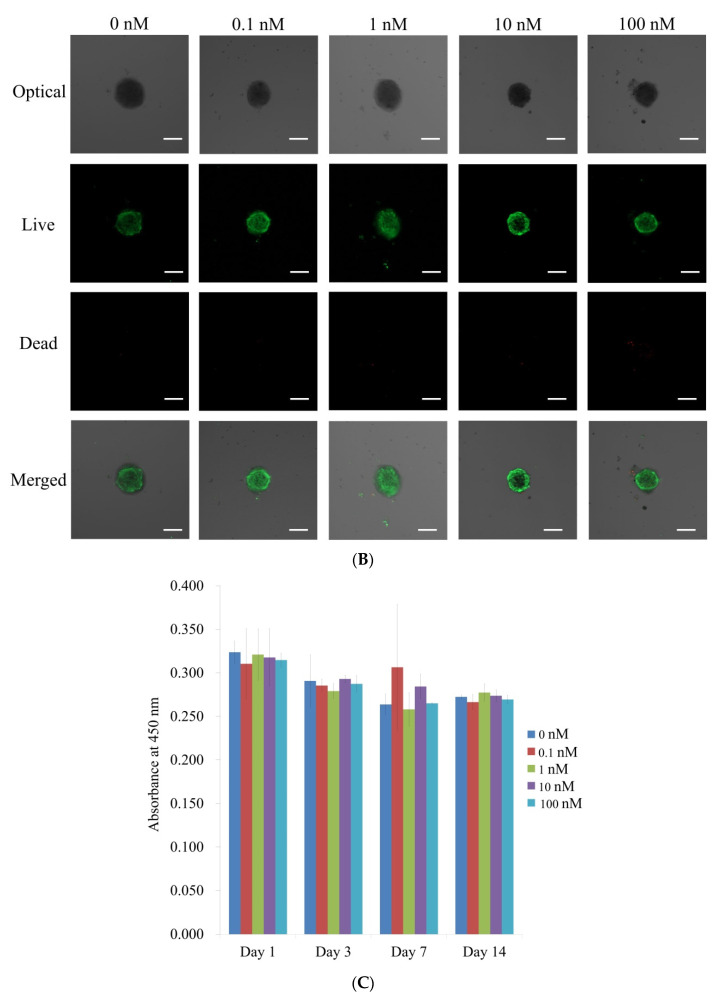
(**A**) Optical, live, dead, and merged cell images of stem cell spheroids on day 1. (**B**) Optical, live, dead, and merged cell images of stem cell spheroids on day 7. The scale bar represents 100 μm (original magnification ×100). (**C**) Cell viability using Cell Counting Kit-8 on days 1, 3, 7, and 14.

The qualitative viability of stem cells was analyzed using a Live/Dead Kit assay on days 1 and 7 (Figure 3A,B). In all cases, we recognized that most of the stem cells showed a round shape with intense green fluorescence, indicating live cells on day 1 (Figure 3A). Longer incubation of cells on day 7 did not show a noticeable decrease in green fluorescence (Figure 3B).

The quantitative cellular viability on days 1, 3, 7, and 14 are shown in Figure 3C. The absorbance values at 450 nm on day 1 were 0.324 ± 0.013, 0.310 ± 0.040, 0.321 ± 0.030, 0.318 ± 0.033, and 0.315 ± 0.008 for vitamin D at 0, 0.1, 1, 10, and 100 nM groups, respectively (*p >* 0.05). On day 3, the absorbance values for vitamin D at 0, 0.1, 1, 10, and 100 nM concentrations were 0.291 ± 0.030, 0.285 ± 0.008, 0.279 ± 0.009, 0.293 ± 0.004, and 0.287 ± 0.010, respectively (*p >* 0.05). On day 7, the absorbance values for vitamin D at 0, 0.1, 1, 10, and 100 nM concentrations were 0.264 ± 0.012, 0.306 ± 0.073, 0.258 ± 0.020, 0.284 ± 0.014, and 0.265 ± 0.001, respectively (*p >* 0.05). The absorbance values at 450 nm on day 14 were 0.272 ± 0.002, 0.266 ± 0.009, 0.277 ± 0.010, 0.274 ± 0.007, and 0.269 ± 0.005 for vitamin D at 0, 0.1, 1, 10, and 100 nM groups, respectively (*p >* 0.05).

### 3.3. Evaluation of Alkaline Phosphatase Activity 

**Figure 4:**
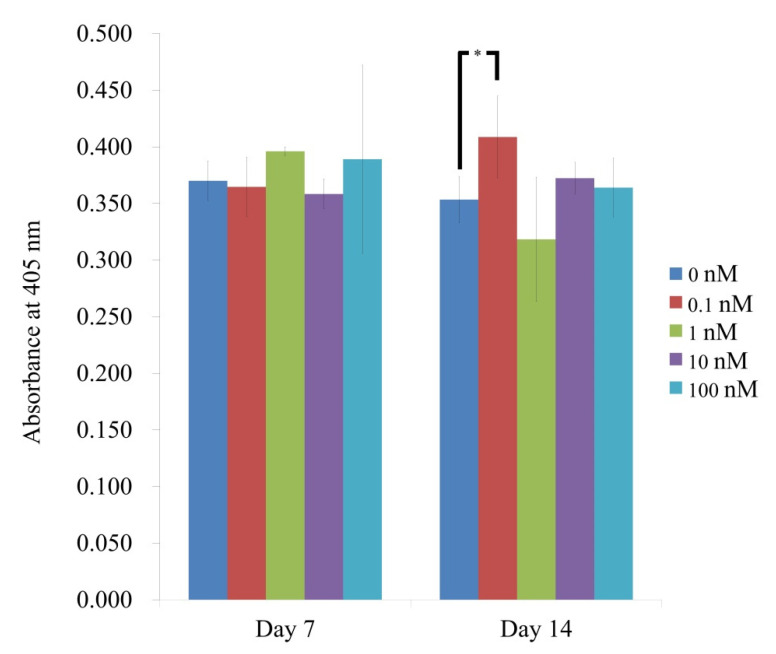
Graphical representation of alkaline phosphatase activity results on days 7 and 14. * *p* < 0.05 vs. time-matched 0 nM group.

The results of alkaline phosphatase activity showed that there was a significant increase in the 0.1 nM group when compared with the control on day 14 (*p* < 0.05) (Figure 4). The absorbance values at 405 nm on day 7 for vitamin D at 0, 0.1, 1, 10, and 100 nM concentrations were 0.370 ± 0.018, 0.365 ± 0.026, 0.396 ± 0.004, 0.358 ± 0.013, and 0.389 ± 0.083, respectively (*p* > 0.05). On day 14, the absorbance values for vitamin D at 0, 0.1, 1, 10, and 100 nM concentrations were 0.353 ± 0.021, 0.409 ± 0.036, 0.318 ± 0.055, 0.372 ± 0.014, and 0.364 ± 0.026, respectively (*p* < 0.05).

### 3.4. Evaluation of RUNX2, BSP, OCN, and COL1A1 by qPCR

**Figure 5:**
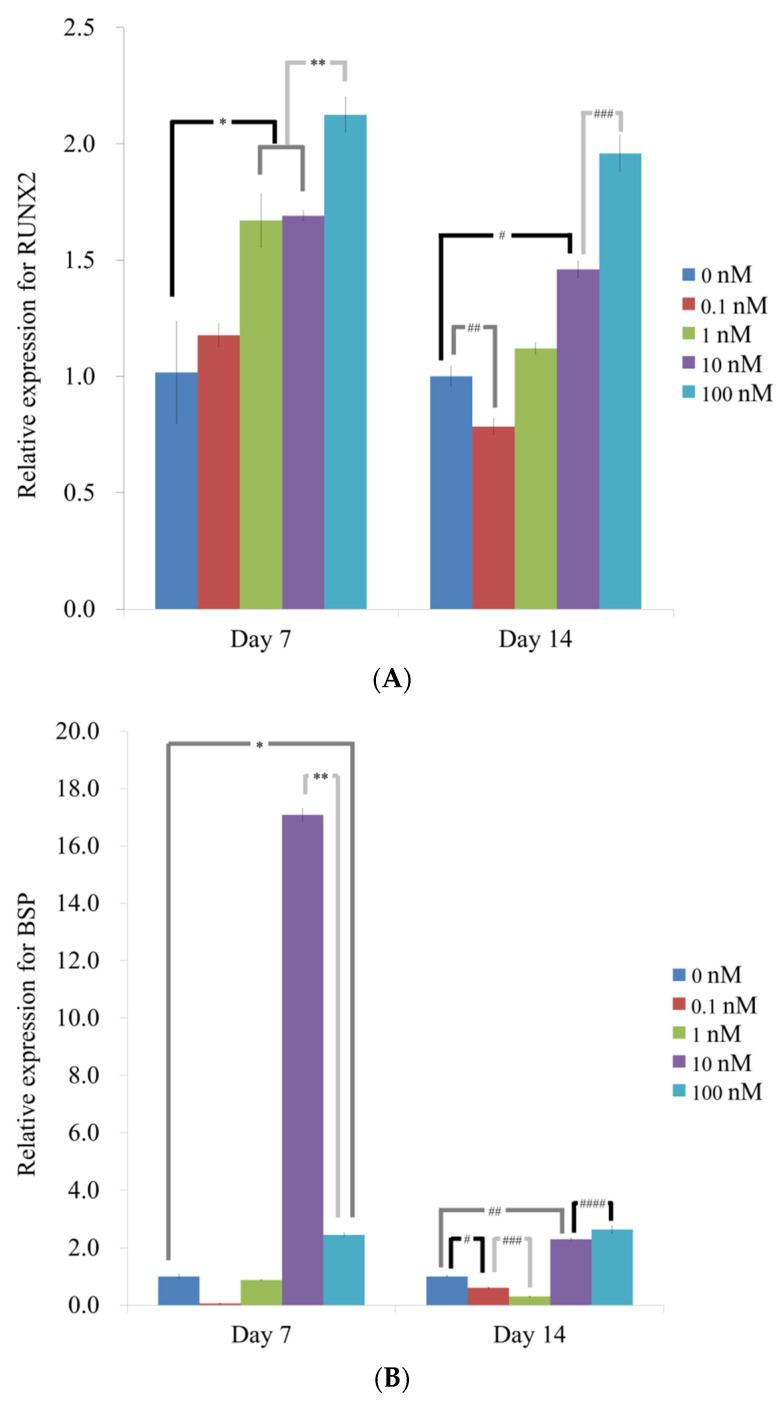

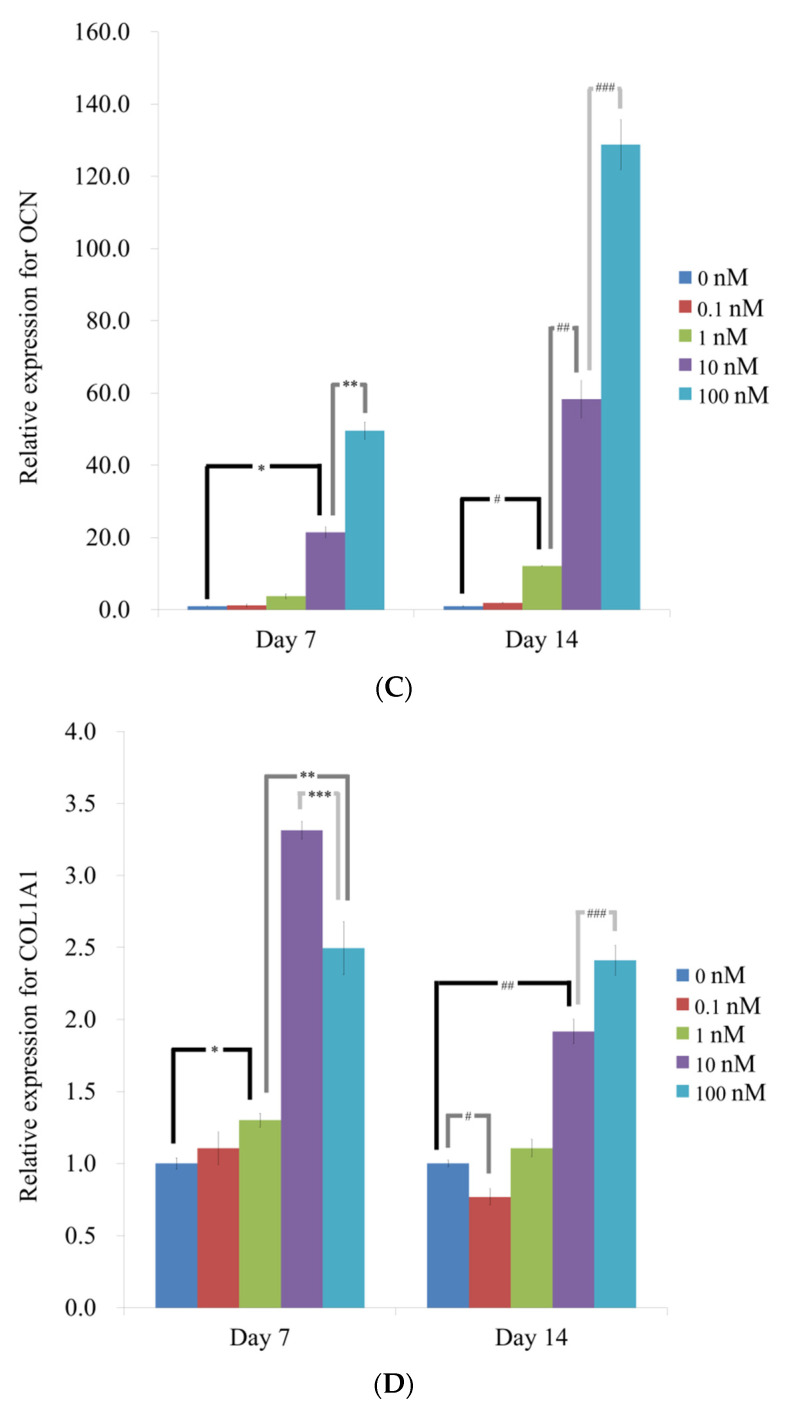
(**A**) Quantification of expression of RUNX2 mRNA by real-time polymerase chain reaction on days 7 and 14. * *p* < 0.05 versus the 0 nM on day 7. ** *p* < 0.05 versus the 1 and 10 nM groups on day 7. ^#^ *p* < 0.05 versus the 0 nM group on day 14. ^##^ *p* < 0.05 versus the 0 nM group on day 14. ^###^ *p* < 0.05 versus the 10 nM group on day 14. (**B**) Quantification of expression of BSP mRNA by real-time polymerase chain reaction on days 7 and 14. * *p* < 0.05 versus the 0 nM on day 7. ** *p* < 0.05 versus the 10 nM group on day 7. ^#^ *p* < 0.05 versus the 0 nM group on day 14. ^##^ *p* < 0.05 versus the 0 nM group on day 14. ^###^ *p* < 0.05 versus the 0.1 nM group on Day 14. ^####^ *p* < 0.05 versus the 10 nM group on day 14. (**C**) Quantification of expression of OCN mRNA by real-time polymerase chain reaction on days 7 and 14. * *p* < 0.05 versus the 0 nM on day 7. ** *p* < 0.05 versus the 10 nM group on day 7. ^#^ *p* < 0.05 versus the 0 nM group on day 14. ^##^ *p* < 0.05 versus the 1 nM group on day 14. ^###^ *p* < 0.05 versus the 10 nM group on day 14. (**D**) Quantification of expression of COL1A1 mRNA by real-time polymerase chain reaction on days 7 and 14. * *p* < 0.05 versus the 0 nM on day 7. ** *p* < 0.05 versus the 1 nM group on day 7. *** *p* < 0.05 versus the 10 nM group on day 7. ^#^ *p* < 0.05 versus the 0 nM group on day 14. ^##^ *p* < 0.05 versus the 0 nM group on day 14. ^###^ *p* < 0.05 versus the 10 nM group on day 14.

qPCR revealed that the mRNA levels of RUNX2 on day 7 were 1.017 ± 0.220, 1.177 ± 0.049, 1.670 ± 0.113, 1.691 ± 0.021, and 2.125 ± 0.074 for vitamin D at 0, 0.1, 1, 10, 100 nM, respectively (*p* < 0.05) (Figure 5A). The addition of vitamin D led to the significant increase in RUNX2 expression at 1, 10 and 100 nM. The results showed that the mRNA levels of RUNX2 on day 14 were 1.001 ± 0.042, 0.785 ± 0.034, 1.121 ± 0.023, 1.460 ± 0.034, and 1.959 ± 0.078 for vitamin D at 0, 0.1, 1, 10, 100 nM, respectively (*p* < 0.05). The addition of vitamin D led to the significant increase in RUNX2 expression at 10 and 100 nM.

qPCR revealed that the mRNA levels of BSP on day 7 were 1.002 ± 0.070, 0.054 ± 0.010, 0.874 ± 0.013, 17.082 ± 0.224, and 2.452 ± 0.076, respectively (*p* < 0.05) (Figure 5B). The addition of vitamin D led to the significant increase in BSP expression at 10 and 100 nM. The results demonstrated that the mRNA levels of BSP on day 14 were 1.000 ± 0.029, 0.600 ± 0.032, 0.300 ± 0.008, 2.300 ± 0.035, and 2.637 ± 0.124, respectively (*p* < 0.05). The addition of vitamin D led to the significant increase in BSP expression at 10 and 100 nM.

qPCR revealed that the mRNA levels of OCN on day 7 were 1.001 ± 0.043, 1.204 ± 0.338, 3.748 ± 0.653, 21.457 ± 1.445, and 49.592 ± 2.344, respectively (*p* < 0.05) (Figure 5C). The addition of vitamin D led to the significant increase in OCN expression at 10 and 100 nM. The results demonstrated that he mRNA levels of BSP on day 14 were 1.006 ± 0.131, 1.888 ± 0.098, 12.125 ± 0.064, 58.288 ± 5.088, and 128.783 ± 6.925, respectively (*p* < 0.05). The addition of vitamin D led to the significant increase in RUNX2 expression at 1, 10 and 100 nM.

qPCR revealed that the mRNA levels of COL1A1 on day 7 were 1.001 ± 0.040, 1.105 ± 0.112, 1.301 ± 0.048, 3.315 ± 0.062, and 2.495 ± 0.184, respectively (*p* < 0.05) (Figure 5D). The addition of vitamin D led to the significant increase in COL1A1 expression at 1, 10 and 100 nM. The results demonstrated that the mRNA levels of COL1A1 on day 14 were 1.000 ± 0.022, 0.770 ± 0.056, 1.107 ± 0.060, 1.917 ± 0.085, and 2.411 ± 0.102, respectively (*p* < 0.05). The addition of vitamin D led to the significant increase in COL1A1 expression at 10 and 100 nM.

## 4. Discussion

This research analyzed the effects of vitamin D on the osteogenic differentiation and mineralization of human mesenchymal stem cells. Differentiation into an osteogenic lineage was detected by alkaline phosphatase activity, and the mRNA levels of RUNX2, BSP, OCN, and COL1A1 were detected using real-time quantitative polymerase chain reaction [26].

Vitamin D has various effects on different tissues and cells [27,28,29,30,31,32,33]. Vitamin D deficiency is reported to result in abnormal calcium, phosphorus and bone metabolism [27]. In particular, vitamin D deficiency reduced the efficiency of intestinal calcium and reduced the absorption of phosphorus from dietary calcium and phosphorus, resulting in increased parathyroid hormone levels [28]. Vitamin D deficiency may impair fracture healing and may worsen bone loss after trauma [29]. A previous report revealed that vitamin D had a regulatory role on human colon stem cells, showing a homeostatic effect on colon epithelium with relevant implications in inflammatory bowel diseases and colorectal cancer [30]. Another previous report showed that vitamin D could be unfavorable in the context of cartilage matrix synthesis [31]. Treatment of vitamin D deficiency led to increase in the level of bone at the implant [32]. There were controversial results regarding bone markers. It was shown that consumption of vitamin D-fortified foods did not show significant changes of bone turnover markers, including osteocalcin and type 1 collagen [33]. 

The effects of different concentrations of vitamin D have been evaluated in previous studies [34,35,36,37]. A previous report showed that MC3T3-E1 osteoblastic cells showed significant increases in alkaline phosphatase activity with vitamin D at 0.1 nM [34]. Treatment with vitamin D at 5 nM and 10 nM led to a statistically significant increase in Alizarin red optical density of mesenchymal stem cells obtained from dental pulp [35]. Human periodontal ligament stem cells were treated with vitamin D at 100 nM and photobiomodulated, and this led to enhanced osteoblastic differentiation [36]. Treatment of primary human skeletal muscle myoblast with vitamin D at 100 nM showed inhibition of myoblast proliferation and enhancement of differentiation [37]. Differences in the maximum effective doses may be due to variability in conditions including cell culture conditions, cell passage, cell type, and incubation time [38,39].

Cellular viability was analyzed with qualitative and quantitative methods [20,40]. Alkaline phosphatase activity is considered as one of the first key players in the process of osteogenesis [41]. Alkaline phosphatase activity has become the marker of choice when assessing the phenotypic or developmental maturity of mineralized tissue cells because of its centrality, biochemical and histological analysis [19]. Expression level of various genes including RUNX2, BSP, OCN, and COL1A1 were studied to analyze the osteogenic potential [42]. RUNX2 is known as a major transcription factor for osteoblasts, and has been widely used for the evaluation of osteogenic differentiation including stem cell spheroids [14,43]. BSP was suggested to be the early marker for osteogenic differentiation of stem cells [44]. The OCN gene encodes protein secreted by osteoblasts that regulates bone remodeling [45]. COL1A1 is also known as an osteogenic marker, and the impairment in collagen formation due to mutation of COL1A1 may lead to fragility of bone [46]. 

Vitamin D was loaded in collagen gel and it was served as an injectable scaffold to accelerated bone growth [47]. Vitamin D was applied with vitamin-conjugated gold nanoparticles as carriers, and they promoted osteogenic differentiation of human adipose-derived stem cells effectively [48]. Previous research reported on the combinatorial use of vitamin D and bone morphogenetic protein 2 [49]. It was also shown that the combination of systemic vitamin D and local Forkhead transcription factor 1 inhibitor can be applied for the enhancement of implant osseointegration [50]. There is some limitation to this study, including the protein expression levels of Runt-related transcription factor 2, bone sialoprotein, osteocalcin and type I collagen.

## 5. Conclusions

This study showed that application of vitamin D had the tendency to increase osteogenic differentiation, as seen from alkaline phosphatase activity and mRNA expression of cell spheroids. Based on these findings, we conclude that vitamin D can be applied for increased osteogenic differentiation of stem cell spheroids. 

## Data Availability

All data analyzed during this study are included in this published article.
